# Radiographic classification and treatment of fibrous dysplasia of the proximal femur: 227 femurs with a mean follow-up of 6 years

**DOI:** 10.1186/s13018-015-0313-6

**Published:** 2015-11-16

**Authors:** Xuelei Zhang, Chunyu Chen, Hong Duan, Chongqi Tu

**Affiliations:** Department of Orthopedics, West China Hospital, Sichuan University, No. 37 Guo Xue Lane, Wuhou District, 610041 Chengdu, Sichuan China

**Keywords:** Fibrous dysplasia, Femur, Radiographic classification, Varus deformity, Valgus osteotomy

## Abstract

**Background:**

Research into the optimal treatment of fibrous dysplasia has been limited by the lack of an established classification system for the disease. The purposes of this study were to develop a radiographic classification for fibrous dysplasia of the proximal femur and to test this classification’s intra- and interobserver reliability as well as the effectiveness of our treatments.

**Methods:**

We retrospectively reviewed radiographs and computed tomography (CT) of 227 femurs from 206 patients with fibrous dysplasia. The radiographs were evaluated in the coronal plane for neck-shaft angle, varus deformity in the proximal femoral shaft, and distal juxtaarticular valgus deformity. CT was evaluated in the axial plane for destruction of cortex. Reduction of bone strength was defined as the thickness of the remaining cortex less than 50 % of the original on axial CT. Two senior orthopedists evaluated each radiograph and CT twice at 8-week intervals. Intra- and interobserver reliability testing was performed using the kappa statistic. Treatments were assessed through mid-term follow-up.

**Results:**

The 227 femurs were classified into five reproducible types: type 1 (33 %), normal bone strength without angular deformity; type 2 (30 %), decreased bone strength without angular deformity; type 3 (12 %), isolated coxa vara with neck-shaft angle <120°; type 4 (11 %), isolated varus deformity in the proximal femoral shaft; and type 5 (14 %), coxa vara with varus deformity in the proximal femoral shaft. Intra- and interobserver kappa values were excellent, ranging from 0.85 to 0.88. Good clinical outcomes were achieved.

**Conclusions:**

This radiographic classification of fibrous dysplasia is reproducible and useful for describing and assessing this disease. The treatments based on this classification were effective.

## Background

Fibrous dysplasia is an uncommon skeletal disorder in which normal bone and bone marrow are replaced by fibro-osseous tissue [1]. It may involve one bone (monostotic fibrous dysplasia) or multiple bones (polyostotic fibrous dysplasia). The proximal femur is a frequent site of fibrous dysplasia, resulting in pain, limping, deformity, and pathological fracture due to the weight of the body and the strong gluteal muscles acting on the weakened proximal femur. The continuous mechanical stress and repeated fractures result in progressive varus and bowing, leading to the classic shepherd’s crook deformity [2]. The descriptions, treatments, and clinical outcomes of similar lesions might significantly differ across studies because there is no established radiographic classification of fibrous dysplasia of the proximal femur to help researchers standardize their understanding and management of this disease [3–10]. In the present study, we classified fibrous dysplasia in the proximal femur into five types through retrospective review of anteroposterior (AP) X-ray films and axial computed tomography (CT) films from 206 patients. We also tested the repeatability of this classification system with the kappa test. We further proposed different treatments according to these varying types and assessed their effectiveness.

## Methods

### Patient information

We searched the medical records of our department (Department of Orthopedics, West China Hospital, China) from January 1995 to January 2013 to identify all patients with fibrous dysplasia in the proximal femur treated in our department during that period. We confirmed 206 patients (227 femurs) who had complete medical records that met all of the following requirements: an AP radiograph of the hip, chart notes with diagnosis, and treatment records of any surgery performed. Of the 206 patients, 97 were males, and 109 were females. Twenty-one had bilateral femoral involvement, 185 had unilateral involvement, 141 had MFD, and 65 had PFD. Ninety-three femurs sustained one to five pathological fractures. The mean follow-up time was 76 months (range, 24–141 months). The mean age at the first visit was 26 years (range, 8–58 years). The mean age at the last follow-up was 35 years (range, 15–63 years). Detailed patient characteristics are shown in Table 1. This study was conducted in accordance with the Declaration of Helsinki and with approval from the Ethics Committee of West China Hospital (Chengdu, China). Written informed consent was obtained from all participants or their guardians.

**Table 1:** Patient characteristics

Variable	Number
Ages (yes)	
<18 years old (%)	42 (26)
>18 years old (%)	164 (74)
Gender (%)	
Female	109 (53)
Male	97 (47)
Types (%)	
Monostotic	141 (68)
Polyostotic	65 (32)
Bilateral femoral involvement	21 (10)
Unilateral femoral involvement	185 (90)
Lesion ranges (%)	
The proximal 1/3 femur only	179 (79)
Distal to the proximal 1/3 femur	48 (21)
Pathological fractures (%)	93 (41)

### Radiographic evaluation

Two senior orthopedists (Tu and Duan) evaluated the femoral radiographs and axial CT twice at 8-week intervals. They were blinded to each other’s results. Fibrous dysplasia was identified by the presence of at least one of the following features: (a) grayish “ground-glass” appearance; (b) endosteal scalloping; (c) shepherd’s crook deformity; and (d) intramedullary, expansible lesion with a smooth sclerotic margin [11]. They evaluated the following parameters: (1) location of fibrous dysplasia lesions within the femur; (2) whether the destruction of the femoral cortex reached 50 % of the original cortical thickness; (3) neck-shaft angle measured on the AP radiograph, defined as the angle between the axis of the femoral neck and the anatomical axis of the femur, or when varus deformity was present in the proximal femur below the lesser trochanter, identified by the intersection of the axis of the femoral neck and the anatomical axis of the trochanteric segment located above the deformity; (4) proximal and distal angular deformity of the femoral shaft, identified in the coronal plane as the angle formed by the axis of the two adjacent segments of the femur encompassing the deformity; and (5) intra- and interobserver agreement for parameters (2), (3), and (4).

### Description of the classification system

**Fig. 1:**
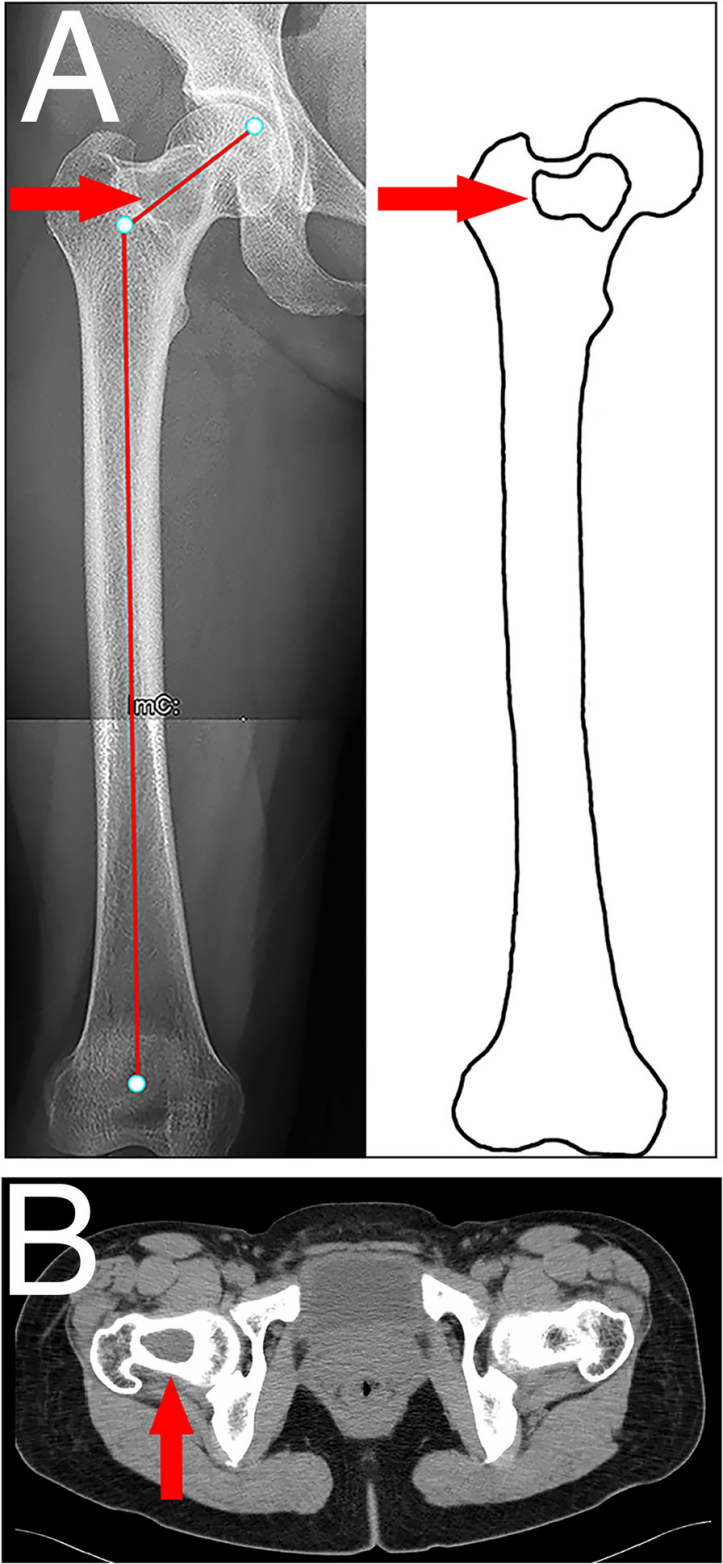
Type 1 femur in a 43-year-old woman. The *red arrow* indicates the lesion. **a** Fibrous dysplasia was present in the femoral neck. The neck-shaft angle was normal (125°). There was no varus deformity in the proximal femoral shaft. **b** The cortex around the lesion was intact on axial CT

**Fig. 2:**
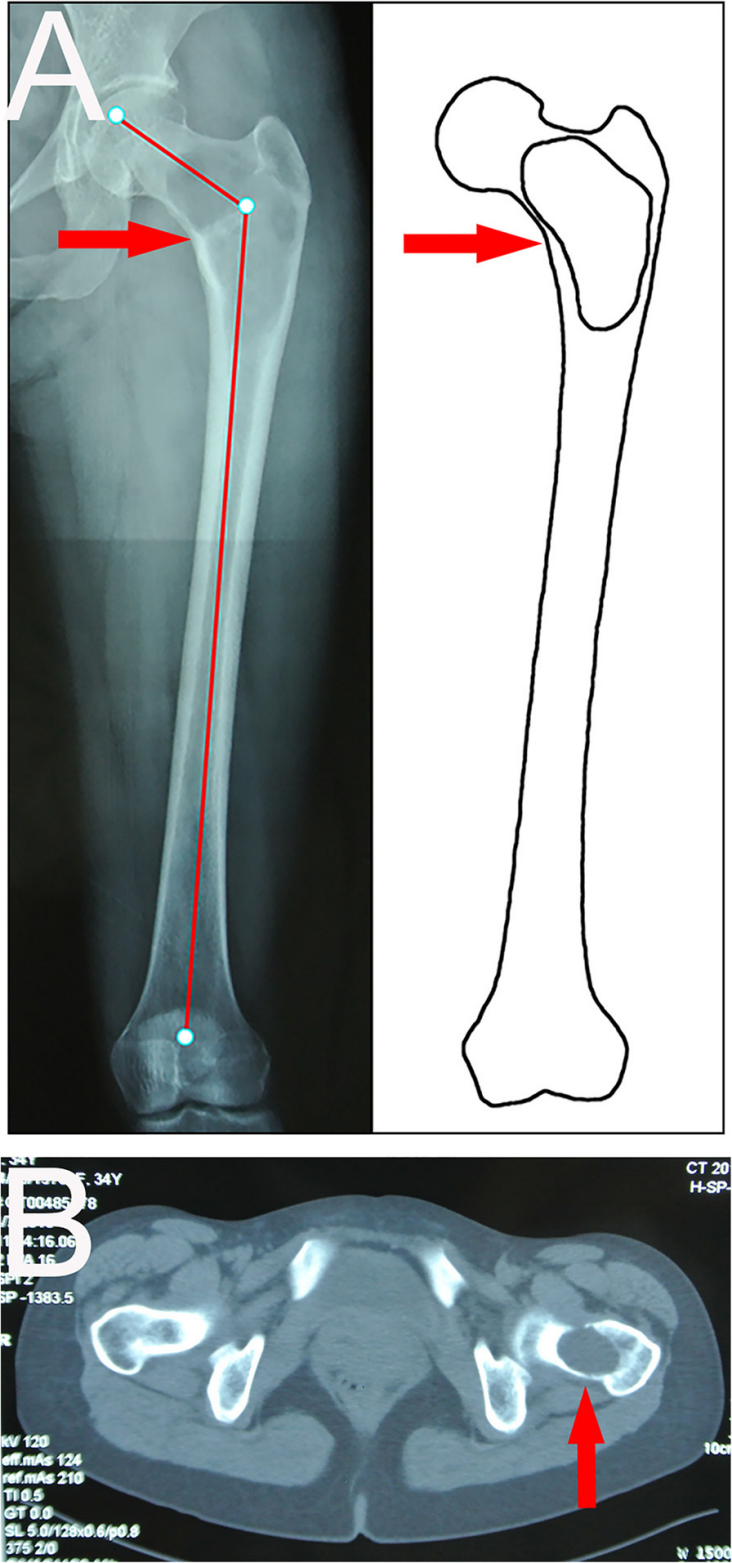
Type 2 lesion in a 35-year-old woman. The *red arrow* indicates the lesion. **a** Fibrous dysplasia was present in the femoral neck and intertrochanteric region. The neck-shaft angle was normal (122°). There was no varus deformity in the proximal femoral shaft. **b** A fracture was present in the intertrochanteric region (the remaining cortex in the intertrochanteric region was less than 50 % of the original thickness)

**Fig. 3:**
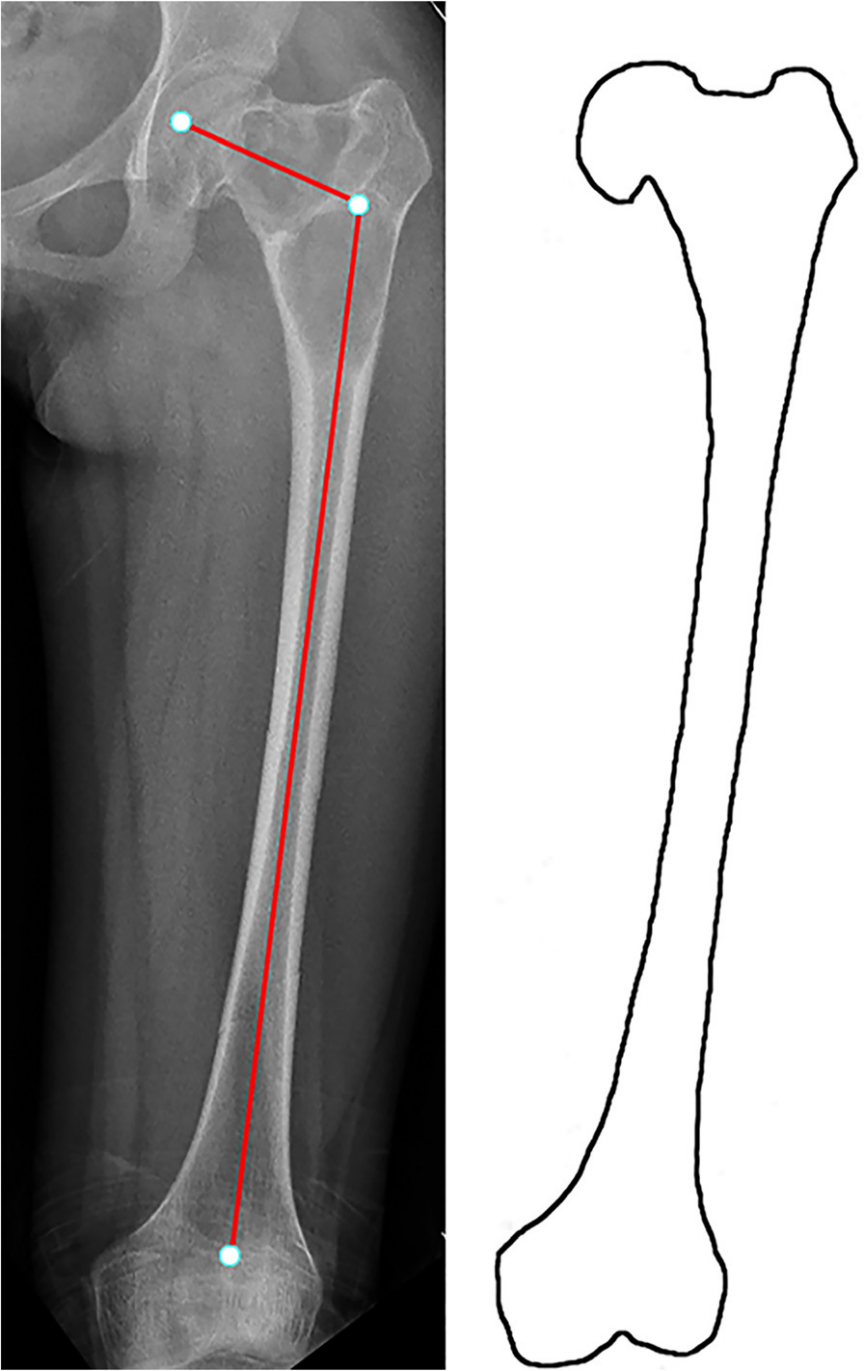
Type 3 lesion in a 15-year-old woman. Fibrous dysplasia was present in the femoral neck and intertrochanteric region. The neck-shaft angle was varus (108°). No varus deformity was present in the proximal femoral shaft. The proximal femoral cortex was thinner than normal

**Fig. 4:**
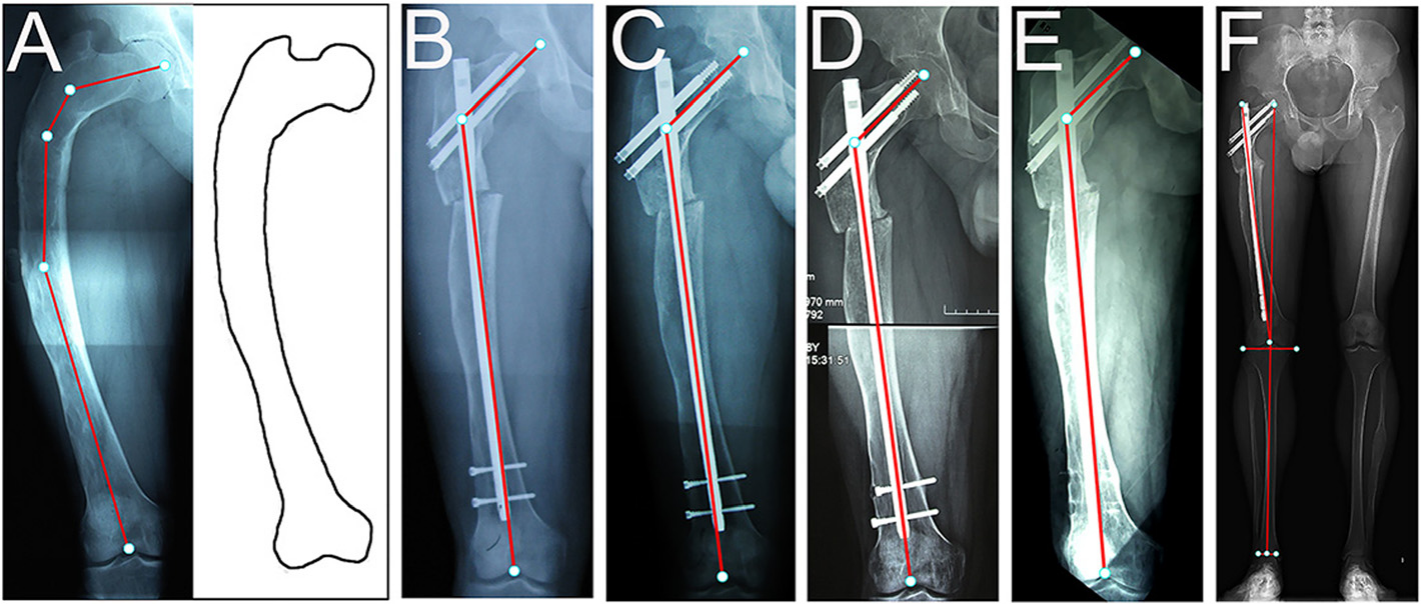
**a** Type 4 lesion in a 28-year-old man. The entire femur was affected by fibrous dysplasia. The neck-shaft angle was 130°. Varus deformity was present in the proximal femoral shaft. The shaft cortex was thinner than normal. **b** The deformity was corrected with single-level valgus osteotomy at the vertex of the deformity. The femur alignment was restored to normal. **c** The osteotomy had not healed 9 months after the operation. **d** The osteotomy had not healed 16 months after the operation. **e** The two distal interlocking screws were removed 16 months after the osteotomy. **f** The osteotomy site healed 9 months after removing the screws. There was no recurrence or progression of the deformity 10 years later

**Fig. 5:**
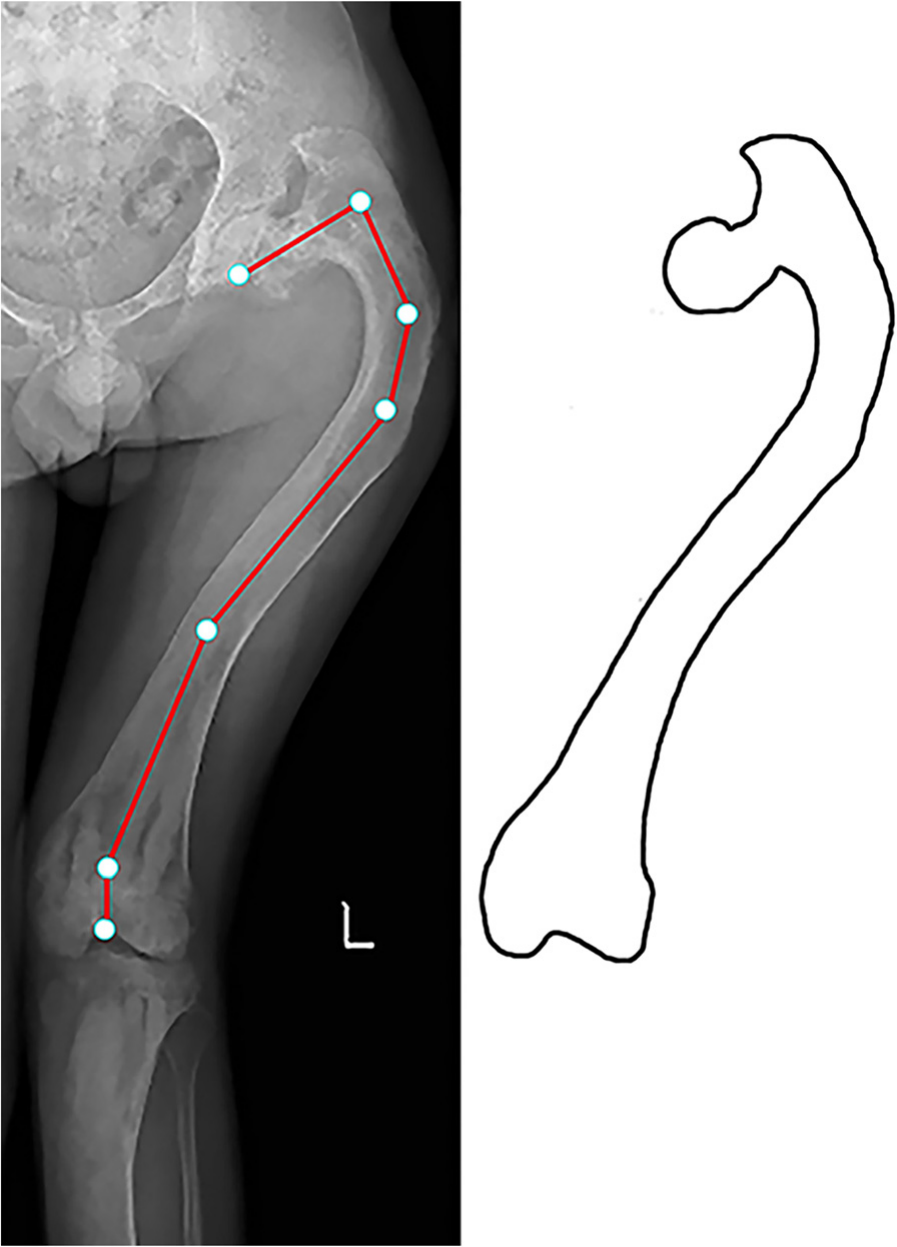
Type 5 lesion in a 27-year-old man. The entire femur was affected by fibrous dysplasia. The neck-shaft angle was 80°. Varus deformity was present in the proximal femoral shaft with juxtaarticular valgus deformity

The 227 femurs were divided into five types according to three parameters: neck-shaft angle, varus deformity of the proximal femoral shaft, and reduction in proximal femoral strength. Reduction in proximal femoral strength was defined as the thickness of the remaining cortical bone <50 % of the original thickness on axial CT. In type 1 and 2 femurs, no coxa vara or varus deformity was present in the proximal femoral shaft. In type 1 femur, the remaining cortical thickness was more than 50 % of the original thickness on axial CT (Fig. 1), while in type 2 femur, the remaining cortical thickness was less than 50 % of the original thickness on axial CT (Fig. 2). In type 3 femur, isolated coxa vara was present with neck-shaft angle <120° (Fig. 3). In type 4 femur, isolated varus deformity was present in the proximal femoral shaft (Fig. 4). In type 5 femur, coxa vara with varus deformity was present in the proximal femoral shaft (Fig. 5). The radiographic classification system is shown in Table 2.

**Table 2:** The radiographic classification system of fibrous dysplasia of the proximal femur

	Parameters		
Types	Coxa vara	Varus deformity in the proximal femoral shaft	Reduction in the proximal femoral strength
Type 1	−	−	−
Type 2	−	−	+
Type 3	+	−	+/−
Type 4	−	+	+/−
Type 5	+	+	+/−

### Statistical analysis: intra- and interobserver agreement

We validated our classification system by calculating the kappa scores for intra- and interobserver agreement. Two senior orthopedists (Tu and Duan) classified each of the 227 femurs into one of the five categories based on AP radiographs and CT and repeated the examination at an 8-week interval. Kappa values >0.75 indicate a high association between two sets of ratings; values between 0.40 and 0.75 indicate a medium association; and those <0.40 indicate a low association.

### Treatment

All 75 type 1 femurs were treated conservatively. Twenty-two patients without pain were treated with regular follow-up only. Fifty-three patients with pain were treated with analgesic to relieve pain and Fosamax to increase bone strength. All 69 type 2 femurs were treated with surgery: 45 femurs with pathological fractures were treated with open reduction and internal fixation, and 24 femurs with reduction in proximal femoral strength were treated with internal fixation alone to provide support for the fragile femurs. In type 3–5 femurs, we tried to correct the deformity with single-level valgus osteotomy to reduce the risk of nonunion of the osteotomy site. We treated type 3 femurs with intertrochanteric or subtrochanteric valgus osteotomy and internal fixation. We treated type 4 femurs with valgus osteotomy at the vertex of the deformity and internal fixation. In type 5 femurs involving coxa vara with varus deformity in the proximal femoral shaft, it was typically difficult to correct the deformities by single-level valgus osteotomy. Therefore, we treated some type 5 femurs with double-level valgus osteotomy combined with internal fixation. One osteotomy was made at the intertrochanteric or subtrochanteric region to correct the coxa vara, and the other was made at the vertex of the varus deformity in the proximal femoral shaft to correct that deformity.

## Results

### Classification system

Seventy-five femurs (33 %) were classified as type 1 with normal bone strength and no deformity in the proximal femur. Sixty-nine femurs (30 %) were classified as type 2 with reduced bone strength and no deformity in the proximal femur. Twenty-seven femurs (12 %) were classified as type 3 with coxa vara only. Twenty-four femurs (11 %) were classified as type 4 with varus deformity in the proximal femoral shaft. Thirty-two femurs (14 %) were classified as type 5 with coxa vara and varus deformity in the proximal femoral shaft. The number of femurs of each type and the locations of the abnormal bone in the different types of femur are shown in Table 3.

**Table 3:** The locations of the abnormal bone in different types of femur

		Lesion locations			
Types		Femoral neck	Femoral trochanter	Neck + trochanter	Trochanter + proximal shaft
Type 1	N1 = 75^a^ (%)	25 (33)	29 (39)	16 (21)	5 (7)
Type 2	N2 = 69 (%)	4 (6)	23 (33)	28 (41)	14 (20)
Type 3	N3 = 27 (%)	0	0	15 (56)	12 (44)
Type 4	N4 = 24 (%)	0	0	0	24 (100)
Type 5	N5 = 32 (%)	0	0	0	32 (100)

^a^There were 75 femurs in type 1

### Intra- and interobserver agreement

The intra- and interobserver agreements were excellent. The kappa score for interobserver agreement was 0.852, and the kappa scores for intraobserver agreement were 0.873 and 0.886.

### Clinical outcomes and progression of the disease

**Fig. 6:**
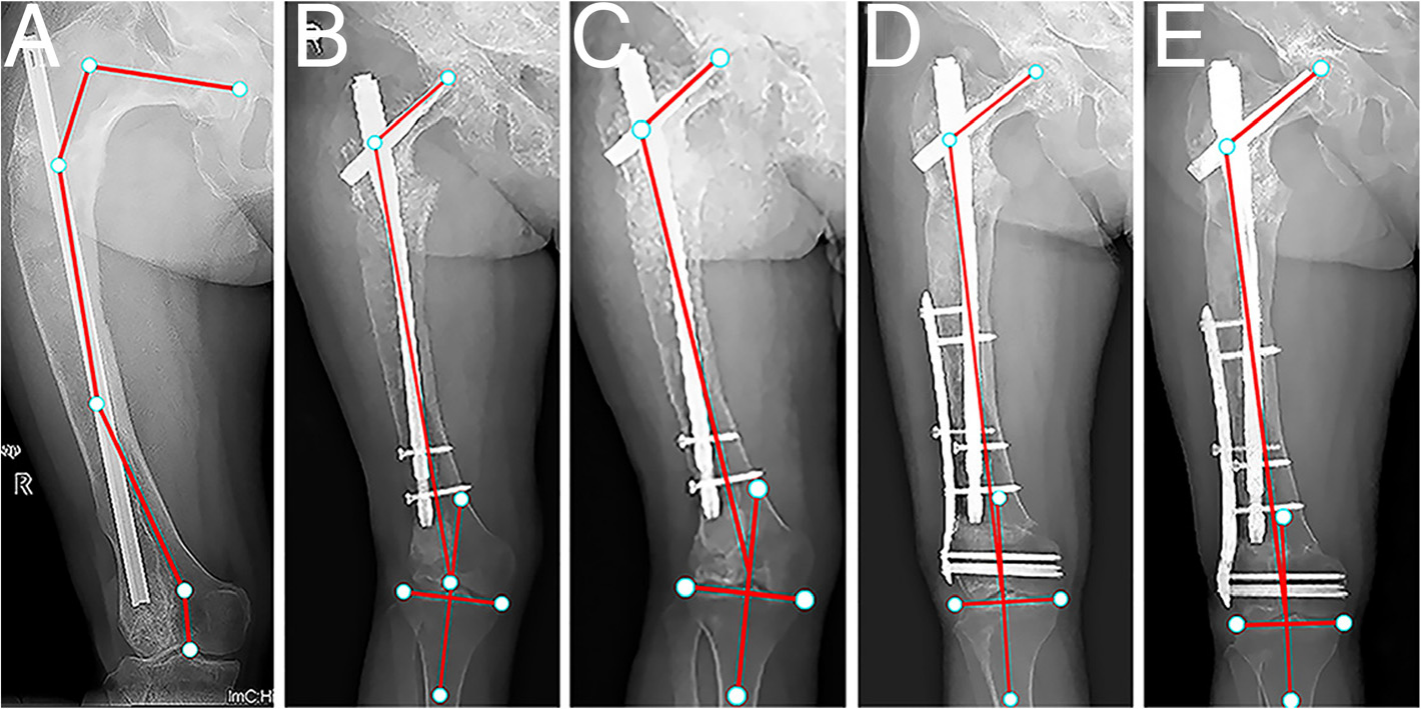
**a** Type 5 lesion in a 36-year-old woman. The entire femur was affected by fibrous dysplasia. The neck-shaft angle was 97°. Coxa vara, varus deformity in the proximal femoral shaft, and juxtaarticular valgus deformity in the distal femur were all present, along with knee osteoarthritis. **b** The coxa vara and varus deformity were corrected with single-level valgus osteotomy made at the vertex of the deformity. The neck-shaft angle was restored to normal (120°). A secondary genu valgum was present with a 20° valgus femorotibial angle. **c** The osteotomy healed 6 months after the operation. **d** A supracondylar femoral varus osteotomy was performed to correct the secondary genu valgum. The femur alignment was restored to normal. **e** A radiograph 96 months after the supracondylar femoral varus osteotomy showed no recurrent deformity

Three type 1 femurs progressed to type 2 and were treated with internal fixation. Two type 2 femurs progressed to type 3, and two progressed to type 5. Forty-five type 2 femurs with fracture treated with open reduction and internal fixation healed 3–6 months after the operations. Three type 3 femurs had recurrence of the deformity. Two type 3 femurs progressed to type 5. Two type 4 femurs had recurrence of the deformity. One type 4 femur progressed to type 5. Three type 5 femurs had recurrence of the deformity. All osteotomy sites of the type 3–5 femurs healed within 6 months after the operation except one type 4 femur (Fig. 4). Additionally, two type 3 femurs and three type 5 femurs had hip osteoarthritis (Fig. 5), and three type 5 femurs had knee joint osteoarthritis (Fig. 6). The details of the clinical outcomes are shown in Table 4.

**Table 4:** The details of the clinical outcomes

		Clinical outcomes				
Types		Pain	Limited hip motion	Limping	Limited daily activities	Limited social activities
Type 1 N1 = 75^a^ (%)	Before treatment	53 (71)	0 (0)	4 (5)	7 (9)	15 (20)
	Post treatment	2 (3)	0 (0)	0 (0)	0 (0)	0 (0)
Type 2 N2 = 69 (%)	Before treatment	62 (90)	24 (35)	54 (78)	56 (81)	58 (84)
	Post treatment	5 (7)	0 (0)	4 (6)	2 (3)	0 (0)
Type 3 N3 = 27 (%)	Before treatment	23 (85)	20 (74)	24 (89)	23 (85)	24 (89)
	Post treatment	4 (15)	4 (15)	1 (4)	1 (4)	3 (11)
Type 4 N4 = 24 (%)	Before treatment	22 (92)	9 (38)	24 (100)	22 (92)	24 (100)
	Post treatment	3 (13)	1 (4)	3 (13)	1 (4)	3 (13)
Type 5 N5 = 32 (%)	Before treatment	32 (100)	15 (47)	32 (100)	32 (100)	32 (100)
	Post treatment	3 (9)	4 (13)	3 (9)	3 (9)	5 (16)

^a^There were 75 femurs in type 1

## Discussion

In reviewing the literature on fibrous dysplasia of the proximal femur, we found that the descriptions, treatments, and outcomes of similar lesions could substantially differ across studies. For example, when treating a femur without fracture and deformity, one can use conservative treatment, curettage and bone grafting, or internal fixation [12, 13]. Therefore, it is difficult for readers to compare the various treatments and choose the appropriate one because there is no such classification system to help standardize the description of this disease. Furthermore, there are no specific treatments based on the different disease types. In this study, we classified fibrous dysplasia of the proximal femur into five reproducible types by retrospectively reviewing AP X-ray and axial CT images of 227 femurs in 206 patients. We also assessed the repeatability of this classification system with the kappa test. Finally, we propose different treatments according to these different types and report the effectiveness of these treatments through mid-term follow-up. We treated 75 type 1 femurs conservatively, and only three type 1 femurs progressed to type 2. Therefore, we believe that conservative treatment may be sufficient for type 1 femurs. DiCaprio and Enneking drew a similar conclusion in a study published in 2005, which was the most comprehensive publication on fibrous dysplasia to date [14]. For type 2 femurs without fracture, internal fixation alone may be sufficient. For type 2 femurs with fracture, open reduction and internal fixation should be performed [15]. For type 3–5 femurs, valgus osteotomy and internal fixation are necessary [5, 6, 8]. For type 5 femurs, double-level osteotomy should be performed to correct the coxa vara and the varus deformity in the proximal femoral shaft. The double-level osteotomy can be performed in a one-stage or two-stage operation [16]. The one-stage valgus osteotomy has several advantages compared with two-stage osteotomy, including shortened treatment time, reduced number of operations, and lower medical costs. Therefore, we treated the 32 type 5 femurs with one-stage double-level valgus osteotomy. All alignments of the lower extremity were corrected to normal. This result is consistent with that of the 2009 report of Liu, who treated five type 5 femurs with double-level valgus osteotomy in a one-stage operation and observed good healing at all osteotomy sites [17].

Finally, type 4 and type 5 femurs may have valgus deformity in the juxtaarticular area, and there may be secondary genu valgum deformity after correcting the varus deformity in the proximal femoral shaft. If the secondary valgus angle was more than 10°, we performed a varus supracondylar osteotomy (Fig. 6). We planned the varus supracondylar osteotomy in the distal femur and the valgus osteotomy in the proximal femur simultaneously before the operation. We performed the varus supracondylar osteotomy just after union of the osteotomy site in the proximal femur. To our knowledge, this study is the first to report the correction of the secondary genu valgum deformity. Our proposed surgical method may help in treating fibrous dysplasia with total femur involvement.

There are several limitations in our study. First, the lesions are described only in the coronal plane. Therefore, the recurrence or progression of the deformity in the sagittal plane could have been underestimated. Further analyses should be based on three-dimensional CT. Second, this study was a retrospective analysis without a control group. These conclusions should be tested with a prospective clinical controlled study in the future. Third, the classification and the treatments are more suitable for adult patients, as the majority of our patients were adults.

## Conclusion

In conclusion, we propose a classification system of fibrous dysplasia of the proximal femur based on the largest cohort of fibrous dysplasia patients reported to date. The high degrees of intra- and interobserver agreement of this classification system indicate its reproducibility. We propose conservative treatment for type 1 femurs, internal fixation or open reduction and internal fixation for type 2 femurs, intertrochanteric or subtrochanteric valgus osteotomy for type 3 femurs, valgus osteotomy performed at the vertex of the deformity for type 4 femurs, and double-level valgus osteotomy for type 5 femurs. In addition, in case of secondary genu valgum, varus supracondylar osteotomy can be performed to restore femur alignment.

## Abbreviations

AP: anteroposterior
CT: computed tomography
